# A note on the relationship between age and health-related quality of life assessment

**DOI:** 10.1007/s11136-018-2071-5

**Published:** 2018-12-06

**Authors:** Patricia Cubi-Molla, Koonal Shah, Jamie Garside, Mike Herdman, Nancy Devlin

**Affiliations:** 1 The Office of Health Economics, London, UK; 2 City, University of London, London, UK; 3 Janssen, High Wycombe, UK

**Keywords:** Time trade-off, Social preferences, EQ-5D profiles, Age-specific value set, HTA decision-making, Age group, Smile plot

## Abstract

**Purpose:**

To extend existing analyses of whether and how the age of respondents is related to their time trade-off (TTO) valuations of hypothetical EQ-5D-3L health states, and to contribute to the existing debate about the rationale and implications for using age-specific utilities in health technology assessment (HTA).

**Methods:**

We use data from the MVH UK valuation study. For each profile, the mean TTO value—adjusted by sex, education, self-reported health and personal experience of serious illness—is pairwise compared across the different age groups. A Bonferroni correction is applied to the multiple testing of significant differences between means. Smile plots illustrate the results. A debate regarding whether there is a case for using age-specific utilities in HTAs complements the analysis.

**Results:**

Results show that the oldest respondents value health profiles lower than younger age groups, particularly for profiles describing problems in the mobility dimension.

**Conclusion:**

The findings raise the possibility of using age-specific value sets in HTAs, since a technology may not be cost-effective on average but cost-effective for a sub-group whose preferences are more closely aligned to the benefits offered by the technology.

**Electronic supplementary material:**

The online version of this article (10.1007/s11136-018-2071-5) contains supplementary material, which is available to authorized users.

## Introduction

In England, the National Institute for Health and Care Excellence (NICE) recommends the use of the MVH^1^ value set to attach utility values to patients’ self-reported EQ-5D-3L profiles in health technology assessments (HTA) [1, 2].^2^ However, some studies have found differences in the valuation of health across groups of people of different ages. These differences could reflect the existence of framing effects linked to the time trade-off (TTO) protocol used in the MVH study [3–5]. Differences may also reflect the different composition of age groups (e.g. older respondents experiencing poorer health) [6]. Finally, individuals of different ages might perceive the same underlying state of health in different ways—mixed evidence has been reported on this [7–9]. If preference heterogeneity across age groups exists and is substantive, then the argument for using general tariffs to inform decisions about the treatment of older people is questionable.

The aim of this research is to (a) extend existing analyses of the relationship between age and utility in the MVH data [2] and (b) contribute to the debate about using age-specific utilities in HTA. We investigate whether and how respondents in different age groups have significantly different valuations for EQ-5D-3L health states. The characteristics of the dataset does not allow us to establish the causal pathway (framing effects, confounding variables, preference heterogeneity) for the observed differences. Nevertheless, our paper extends the previous literature in two ways. First, we improve the level of detail of the analysis by introducing six age groups, exploring the utility differences by health profile. Second, to our knowledge, our paper is the first to apply a robust methodology to identify and graphically depict significant differences in health valuations by age group in the MVH value set.

## Data and methodology

We use the data collected in 1993 for the MVH study to generate a UK value set for the EQ-5D [10]. The MVH study elicited preferences from 2997 members of the general population^3^ regarding 12 health profiles for each individual (35,964 observations) from 42 hypothetical EQ-5D health states (selected from the 243 possible profiles), following the TTO protocol. The analysis is performed over the re-scaled (from − 1 to 1) TTO values, for each of the 42 EQ-5D-3L profiles [2]. We define six age intervals: 18–27 years (*n* = 5568); 28–37 years (*n* = 8100); 38–47 years (*n* = 6156); 48–57 years (*n* = 4680); 58–67 years (*n* = 5064); 68+ years (*n* = 6372).^4^

We first test for the existence of significant differences between means for age groups (null hypothesis: equal means), for each profile. A Bonferroni correction is applied to adjust for multiple testing.^5^ The age-related differences identified are then explored in more detail. For each profile, TTO values are regressed^6^ over age groups, controlling for sex, self-reported health state (level sum score), education and personal experience with serious illness. The adjusted mean TTO value is pairwise compared across the different age groups, by testing for the significance of the age-related coefficients. A Bonferroni correction is also applied here to adjust for multiple testing. Three “smile plots” are used to illustrate the results [13]. Smile plots are a useful tool to summarize the results from a set of multiple tests, helping to separate (by a parapet^7^ line) null hypotheses that are rejected from those that cannot be rejected, at different *p*-values. The graphs also give quantitative information, at a glance, about the estimated parameters being tested. Smile plots have been used (yet not extensively) in medicine, epidemiology and genetics, but up to our knowledge, our paper is the first one using this tool in a health economics setting. We plot data points corresponding to every Bonferroni-corrected pairwise test, with the statistical significance (*p*-value of the test) on the *y*-axis and the average difference in TTO values between the corresponding age categories on the *x*-axis.^8^

## Results

Fifteen significance tests (pairwise comparison over six age groups) are conducted for each health profile.^9^ Results suggest that the oldest age group (68+) is the one that most frequently shows differences in TTO values, compared to the average values in other age groups. These differences are more likely to be observed in moderate and severe health states, in particular in profiles with level 3 in mobility or level 2 or 3 in self-care.

**Fig. 1:**
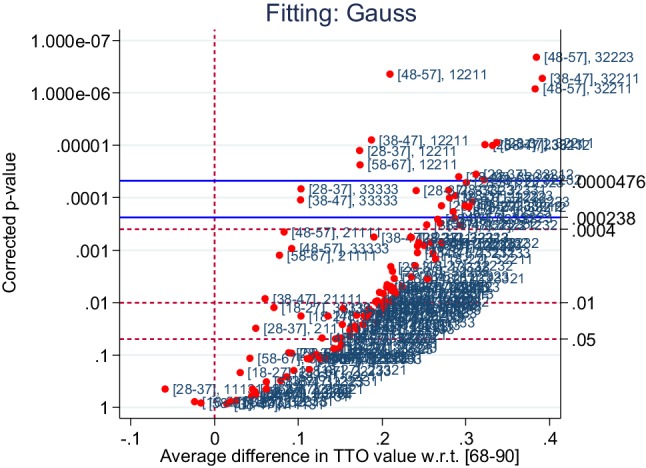
Smile plot showing average differences (*x*-axis) and *p*-values (*y*-axis) for every pairwise comparison of mean TTO values amongst age groups, for several EQ-5D-3L health profiles. Regression based on generalised lineal model, assuming Gaussian distribution. *p*-values represented on a log scale. Age group 68+ as reference. Health profiles represented here: 11131, 11133, 12211, 12223, 13332, 21111, 21133, 21232, 21323, 22121, 22233, 22323, 22331, 23232, 23313, 23321, 32211, 32223, 32232, 32313, 32331, 33212, 33321, 33323, 33333

**Fig. 2:**
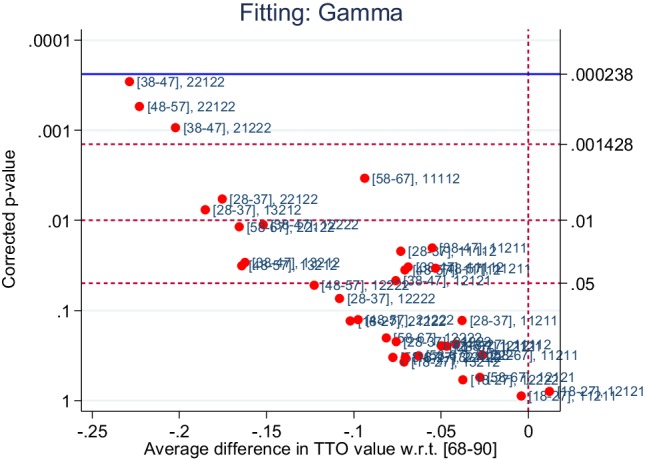
Smile plot showing average differences (*x*-axis) and *p*-values (*y*-axis) for every pairwise comparison of mean TTO values amongst age groups, for several EQ-5D-3L health profiles. Regression based on generalised lineal model, assuming Gamma distribution. *p*-values represented on a log scale. The dependent variable used is 1 − TTO, so negative values imply higher valuations in other age groups compared to 68+. Age group 68+ as reference. Health profiles represented here: 11112, 11211, 12121, 12222, 13212, 21222, 22122

**Fig. 3:**
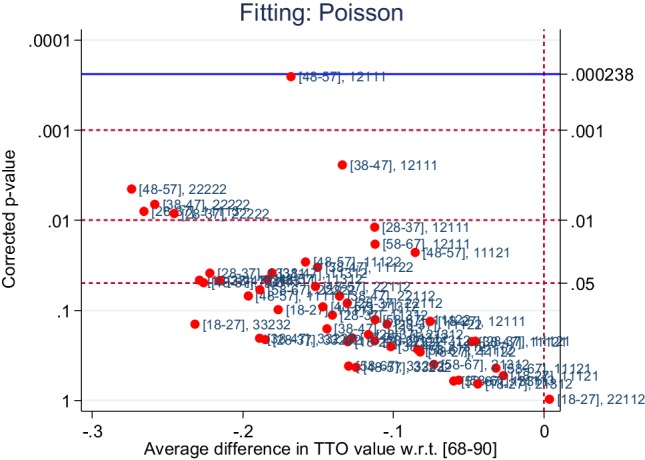
Smile plot showing average differences (*x*-axis) and *p*-values (*y*-axis) for every pairwise comparison of mean TTO values amongst age groups, for several EQ-5D-3L health profiles. Regression based on generalised lineal model, assuming Poisson distribution. *p*-values represented on a log scale. Age group 68+ as reference. The dependent variable used is 1 − TTO, so negative values imply higher valuations in other age groups compared to 68+. Health profiles represented here: 11113, 11121, 11122, 11312, 12111, 13311, 21312, 22112, 22222, 33232

Health profiles are split into three groups for regression analysis, depending on the best fitting model. We test the significance of the regression coefficients for five age groups (68+ is used as a reference group), applying Bonferroni’s correction.^10^ The results from the tests are illustrated using three smile plots [13] in Fig. 1 (Gauss fitting), Fig. 2 (Gamma fitting), and Fig. 3 (Poisson fitting). Each dot in the figures refers to the result of one of the multiple comparison tests, as indicated in the label. For instance, the upper triangle in Fig. 1 (labelled “[48–57], 32,223”) refers to the test comparing the average TTO value attached to profile 32223 obtained from the age groups 48–57 and 68+ (the null hypothesis is that of equality of the means), controlling for the potential effect of other variables as education, sex, and health experience. The coordinates are approximately (0.38, 1.0e−6.8), i.e. with a probability of almost 1, the average TTO value for profile 32223 obtained from 48 to 57 years-old is 0.38 points higher than the average reported by those aged 68 or above.

Dots above the solid parapet lines represent differences which are statistically significant with a confidence level of 95% and 99%. The data points captured between the solid lines and the *y*-axis reference lines for 0.05 and 0.01 represent the pairs of age groups and profiles for which we would have found significant differences, had the uncorrected *p*-values of 0.05 or 0.01 been used for each test.^11^

Table 1 shows the set of health profiles for which the TTO values provided by the oldest respondents are significantly lower than those provided by the other groups, at the 99% level of significance. Note that the differences are such that we find scenarios like the one described for the health state 32211, considered (on average) worse than dead by the oldest respondents and better than dead by younger respondent groups. Also, the value differences can be as large as 0.40 (minimum clinically important difference are usually in the range [0.03, 0.54]) [14], and, therefore, the dissimilarities in the utility values can be of relevance. Probit regressions indicate that differences^12^ are more likely to be significant for profiles describing problems in mobility.

**Table 1:** Average TTO value by age group, for the health states associated to statistically significant differences (99%)

	18–27	28–37	38–47	48–57	58–67	68+
12211	0.7356	*0.8006*	*0.8119*	*0.8341*	*0.8054*	0.6279
23313	− 0.0535	*0.0463*	− 0.0807	− 0.0808	− 0.0396	− 0.2329
32211	0.1634	*0.2266*	*0.2876*	*0.2850*	0.0009	− 0.0806
32223	− 0.2076	− 0.1182	− 0.0836	*0.0087*	− 0.2418	− 0.3810
32232	− 0.1765	− 0.2016	− 0.1344	− *0.0905*	− 0.2857	− 0.4059
32331	− 0.3081	− 0.1753	− *0.1677*	− 0.1970	− 0.3688	− 0.4718
33212	0.0484	*0.0644*	*0.0864*	0.0549	− 0.1397	− 0.2490

In italics, average TTO value associated to rejected null hypothesis. Reference group used for the regression analysis: 68+

## Discussion

We have found that the oldest respondents in the MVH dataset value health profiles lower than younger respondents.^13^ This finding contributes to the debate about whether there is a case for using age-specific utilities in HTAs.

In an ideal world, the preferences of *all* individuals in society could be known and judgements about the cost-effectiveness of a given technology could be made for each person individually. However, this is clearly infeasible, so the average preferences of a sample of the public are used to infer the preferences of wider society. Sculpher and Gafni [15] liken this to searching for a figurative ‘representative individual’, when in fact such an individual cannot exist due to the considerable heterogeneity in people’s preferences. Further, the correct way of ‘averaging’ preference data is unclear [16] and the average of all observed preferences is not the same as the preference of the average person. The use of average preferences, combined with an appropriate decision rule, results in the technology “either being considered ‘cost-effective’ or ‘not cost-effective’ *for all individuals* regardless of the variation between individuals which underlies the average preferences” [15]. Ignoring heterogeneity thus results in a suboptimal use of scarce heath care resources.

Sculpher and Gafni [15] argue that *preference* sub-group analysis can recognise that there may exist a sub-group of a population whose preferences are sufficiently different to the whole-group average so as to produce qualitatively different incremental cost-effectiveness ratios. This opens up the possibility that a technology is cost-*ineffective* on average but cost-*effective* for a sub-group whose preferences over health are more closely aligned to the benefits offered by the technology (see Robinson and Parkin [17] for a critique of this position).

UK health care decision makers are required to respect anti-discrimination legislation that states that patients must not be denied (or have restricted) access to NHS care because of their age. This suggests that it would not be acceptable to use age as a basis for defining sub-groups if this results in denying patients access to treatment based solely on their age. On the other hand, NICE’s *Social Value Judgements* guide notes that its guidance might be able to refer to age if, amongst other things, “there is good evidence, or good grounds, for believing that because of their age patients will respond differently to the treatment in question” [18]. In other words, age-based sub-groups are acceptable if they are *clinically relevant*.

To give an extreme hypothetical example, suppose there was evidence that people aged under 68 years placed *very little weight* on improvements in mobility whereas people aged 68 years and over placed *very much importance* on this dimension. This suggests that a treatment whose primary clinical effect is to improve the patient’s ability to walk about would generate substantial health benefits for the older sub-group but few benefits for the younger sub-group. These health benefits could be expressed in terms of QALYs—treating older patients would result in a large QALY gain whereas treating younger patients would not. It is unclear whether such a case would be interpreted by NICE as demonstrating relevant differences in effects across age groups, and, therefore, whether age-specific guidance in relation to this treatment would be justified.

## Electronic supplementary material

Below is the link to the electronic supplementary material.

Supplementary material 1 (DOCX 57 KB)

## Footnotes

1 Measurement and valuation of health.

2 Although a five-level version of the EQ-5D—the EQ-5D-5L—is now available, at the time of writing NICE continues to recommend use of the EQ-5D-3L/MVH value set.

3 Corresponds to the final sample size used for the MVH report (with an initial sample of 3395 respondents). We have used the same exclusion criteria as the MVH Group [10].

4 Further details about the sample composition (age distribution of respondents, sample size and average TTO value by age group and health profile) are available in supplementary appendix.

5 Bonferroni is a rather conservative procedure, potentially leading to a higher rate of “false negatives” (not detecting actual differences between groups) than “false positives” (detecting non-existing differences between age groups) [11].

6 Best fitting generalised linear models (Gaussian, Gamma and Poisson) according to a Modified Park Test [12] were used for this purpose.

7 The reference line is named ‘parapet line’ because, using a quote from the authors, “null hypotheses that raise their heads above it are shot down” [13].

8 The *x*-axis in Smile plots assigned to Gamma and Poisson regression models show a function of these differences, so that the figures should not be interpreted as linear differences in TTO values.

9 The Bonferroni-corrected overall critical *p*-values are *p* = 0.05/(15 * 42) = 0.0000794 and *p* = 0.0000159 for 95% and 99% level of statistical significance, respectively. Test results not shown here.

10 Corrected *p*-value cut-off: 0.000238 and 0.0000476 for 95% and 99% level of confidence, respectively.

11 Note that these data points could also show true significant differences amongst age groups, but we cannot separate them from the expected 5% or 1% of associations found “by chance” (“false positives”).

12 At the 99% and 95% level of significance.

13 Details of TTO values by age group and health profile can be found in Table S2 in supplementary appendix.
