# Supplementary material for: A note on the relationship between age and health-related quality of life assessment
Source: Qual Life Res. 2018 Dec 6;28(5):1201–5. doi: 10.1007/s11136-018-2071-5 (PMC6470117; doi:10.1007/s11136-018-2071-5)
Supplement: Supplementary file 1 — Supplementary material 1 (DOCX 57 KB) [file 11136_2018_2071_MOESM1_ESM.docx]

**SUPPLEMENTARY MATERIAL**

**1. Age groups and age distribution**

We define five 10-year age intervals: 18-27 years-old, 28-37 years-old, 38-47 years-old, 48-57 years-old, and 58-67 years; and a group embracing respondents 68 years-old and above (max: 90). Table S1 shows details about the sample size of the groups.

Figure S1 illustrates the distribution of ages for all the survey respondents. Age groups are marked with vertical lines.

**Figure S1**: distribution of ages for all the survey respondents


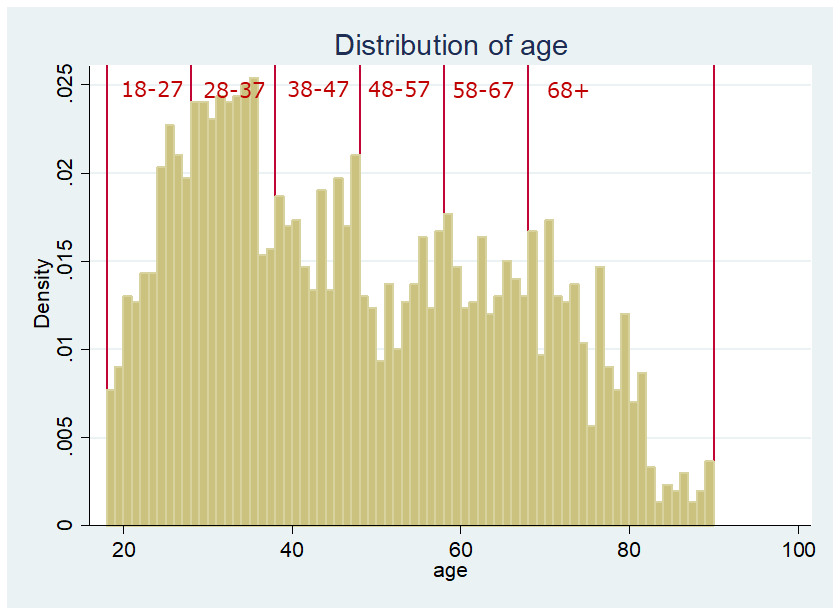


**2. Descriptive statistics**

Table S1 shows the frequencies for respondents valuing hypothetical health states, by age group. Table S2 shows the average TTO value by health state and age group, for each health profiles included in the MVH survey.

**Table S1**: frequencies for respondents valuing hypothetical health states, by age group.

|  | Age group | | | | | |  |
| --- | --- | --- | --- | --- | --- | --- | --- |
| profile | 18-27 | 28-37 | 38-47 | 48-57 | 58-67 | 68-90 | **TOTAL** |
| 11112 | 198 | 262 | 217 | 161 | 171 | 198 | **1,207** |
| 11113 | 128 | 146 | 135 | 87 | 111 | 146 | **753** |
| 11121 | 183 | 270 | 191 | 150 | 175 | 236 | **1,205** |
| 11122 | 123 | 170 | 128 | 96 | 102 | 119 | **738** |
| 11131 | 122 | 196 | 117 | 96 | 93 | 106 | **730** |
| 11133 | 116 | 189 | 132 | 93 | 96 | 129 | **755** |
| 11211 | 175 | 285 | 203 | 154 | 190 | 202 | **1,209** |
| 11312 | 110 | 153 | 139 | 105 | 108 | 133 | **748** |
| 12111 | 189 | 279 | 212 | 150 | 161 | 203 | **1,194** |
| 12121 | 126 | 187 | 128 | 103 | 92 | 136 | **772** |
| 12211 | 114 | 170 | 118 | 107 | 105 | 136 | **750** |
| 12222 | 107 | 176 | 139 | 89 | 106 | 130 | **747** |
| 12223 | 119 | 169 | 137 | 100 | 108 | 121 | **754** |
| 13212 | 118 | 177 | 124 | 99 | 101 | 125 | **744** |
| 13311 | 125 | 157 | 124 | 94 | 101 | 139 | **740** |
| 13332 | 104 | 171 | 117 | 104 | 109 | 131 | **736** |
| 21111 | 182 | 254 | 204 | 165 | 147 | 223 | **1,175** |
| 21133 | 104 | 178 | 131 | 92 | 104 | 143 | **752** |
| 21222 | 117 | 155 | 135 | 99 | 106 | 127 | **739** |
| 21232 | 120 | 179 | 132 | 92 | 103 | 138 | **764** |
| 21312 | 106 | 146 | 110 | 100 | 116 | 144 | **722** |
| 21323 | 112 | 157 | 147 | 90 | 101 | 140 | **747** |
| 22112 | 110 | 152 | 147 | 94 | 130 | 128 | **761** |
| 22121 | 115 | 184 | 118 | 96 | 103 | 145 | **761** |
| 22122 | 113 | 165 | 119 | 87 | 114 | 143 | **741** |
| 22222 | 110 | 191 | 125 | 112 | 103 | 129 | **770** |
| 22233 | 131 | 173 | 109 | 112 | 112 | 124 | **761** |
| 22323 | 112 | 183 | 138 | 97 | 96 | 117 | **743** |
| 22331 | 120 | 167 | 125 | 107 | 88 | 132 | **739** |
| 23232 | 111 | 146 | 129 | 103 | 100 | 136 | **725** |
| 23313 | 115 | 174 | 115 | 95 | 109 | 142 | **750** |
| 23321 | 119 | 165 | 150 | 88 | 99 | 128 | **749** |
| 32211 | 112 | 165 | 125 | 110 | 111 | 122 | **745** |
| 32223 | 122 | 169 | 128 | 98 | 98 | 133 | **748** |
| 32232 | 114 | 170 | 128 | 87 | 115 | 135 | **749** |
| 32313 | 127 | 164 | 115 | 85 | 113 | 154 | **758** |
| 32331 | 111 | 158 | 126 | 106 | 117 | 121 | **739** |
| 33212 | 125 | 154 | 132 | 102 | 124 | 130 | **767** |
| 33232 | 118 | 165 | 133 | 103 | 95 | 142 | **756** |
| 33321 | 112 | 175 | 128 | 86 | 104 | 136 | **741** |
| 33323 | 109 | 179 | 133 | 96 | 105 | 139 | **761** |
| 33333 | 464 | 675 | 513 | 390 | 422 | 531 | **2,995** |
| **TOTAL** | **5,568** | **8,100** | **6,156** | **4,680** | **5,064** | **6,372** | **35,940** |

**Table S2**: average TTO value by health state and age group.

|  | Age group | | | | | |
| --- | --- | --- | --- | --- | --- | --- |
| profile | 18-27 | 28-37 | 38-47 | 48-57 | 58-67 | 68-90 |
| 11112 | 0.8195 | 0.8499 | 0.8360 | 0.8463 | 0.8539 | 0.7648 |
| 11113 | 0.3928 | 0.4885 | 0.3783 | 0.4685 | 0.3365 | 0.3064 |
| 11121 | 0.8318 | 0.8570 | 0.8572 | 0.9016 | 0.8441 | 0.8202 |
| 11122 | 0.6961 | 0.7169 | 0.7705 | 0.7731 | 0.7579 | 0.6310 |
| 11131 | 0.1754 | 0.1476 | 0.2218 | 0.2521 | 0.2542 | 0.2146 |
| 11133 | 0.0125 | -0.0022 | -0.0434 | -0.0540 | -0.0052 | -0.2048 |
| 11211 | 0.8468 | 0.8841 | 0.8940 | 0.8928 | 0.8602 | 0.8342 |
| 11312 | 0.5415 | 0.5588 | 0.6020 | 0.6090 | 0.5649 | 0.4500 |
| 12111 | 0.8178 | 0.8521 | 0.8677 | 0.8973 | 0.8464 | 0.7331 |
| 12121 | 0.7128 | 0.7644 | 0.7863 | 0.7621 | 0.7175 | 0.6999 |
| 12211 | 0.7356 | 0.8006 | 0.8119 | 0.8341 | 0.8054 | 0.6279 |
| 12222 | 0.5017 | 0.5841 | 0.6212 | 0.6025 | 0.5285 | 0.4524 |
| 12223 | 0.3075 | 0.2753 | 0.1897 | 0.2855 | 0.1878 | 0.0496 |
| 13212 | 0.3438 | 0.4609 | 0.4366 | 0.4404 | 0.3501 | 0.2774 |
| 13311 | 0.4082 | 0.4099 | 0.4087 | 0.4093 | 0.2459 | 0.1926 |
| 13332 | -0.1899 | -0.1633 | -0.1735 | -0.1284 | -0.3326 | -0.3807 |
| 21111 | 0.8593 | 0.8779 | 0.8909 | 0.9145 | 0.9105 | 0.8321 |
| 21133 | -0.0469 | -0.0166 | -0.0466 | 0.0399 | -0.0599 | -0.2164 |
| 21222 | 0.5409 | 0.5351 | 0.6315 | 0.6014 | 0.5391 | 0.4759 |
| 21232 | 0.0531 | 0.0620 | 0.1261 | 0.1705 | 0.0889 | -0.0723 |
| 21312 | 0.4794 | 0.5507 | 0.5583 | 0.6088 | 0.5552 | 0.4840 |
| 21323 | 0.1297 | 0.1901 | 0.1913 | 0.2208 | 0.2178 | 0.0355 |
| 22112 | 0.5739 | 0.7140 | 0.7190 | 0.7257 | 0.6628 | 0.5784 |
| 22121 | 0.5973 | 0.6363 | 0.7393 | 0.6904 | 0.6549 | 0.5648 |
| 22122 | 0.4879 | 0.5930 | 0.6142 | 0.6221 | 0.5602 | 0.3901 |
| 22222 | 0.4424 | 0.5500 | 0.5718 | 0.5814 | 0.5084 | 0.3270 |
| 22233 | -0.1536 | -0.0615 | -0.1293 | -0.0602 | -0.1969 | -0.2714 |
| 22323 | -0.0163 | 0.1400 | 0.1047 | 0.0766 | 0.0586 | -0.1709 |
| 22331 | 0.0198 | 0.0431 | -0.0241 | -0.0806 | 0.0665 | -0.0877 |
| 23232 | -0.0113 | -0.0230 | 0.0081 | -0.0434 | -0.1880 | -0.2463 |
| 23313 | -0.0535 | 0.0463 | -0.0807 | -0.0808 | -0.0396 | -0.2329 |
| 23321 | 0.1479 | 0.1895 | 0.2158 | 0.2795 | 0.0515 | -0.0084 |
| 32211 | 0.1634 | 0.2266 | 0.2876 | 0.2850 | 0.0009 | -0.0806 |
| 32223 | -0.2076 | -0.1182 | -0.0836 | 0.0087 | -0.2418 | -0.3810 |
| 32232 | -0.1765 | -0.2016 | -0.1344 | -0.0905 | -0.2857 | -0.4059 |
| 32313 | -0.1813 | -0.0559 | -0.0559 | -0.0565 | -0.2569 | -0.2705 |
| 32331 | -0.3081 | -0.1753 | -0.1677 | -0.1970 | -0.3688 | -0.4718 |
| 33212 | 0.0484 | 0.0644 | 0.0864 | 0.0549 | -0.1397 | -0.2490 |
| 33232 | -0.2282 | -0.2802 | -0.2923 | -0.3612 | -0.3539 | -0.4757 |
| 33321 | -0.0864 | -0.0549 | -0.0270 | -0.1128 | -0.2375 | -0.2325 |
| 33323 | -0.3280 | -0.2634 | -0.3479 | -0.3987 | -0.4779 | -0.5491 |
| 33333 | -0.5391 | -0.5067 | -0.5091 | -0.5211 | -0.5778 | -0.6164 |
